# Cell wall composition in *Cryptococcus neoformans* is media dependent and alters host response, inducing protective immunity

**DOI:** 10.3389/ffunb.2023.1183291

**Published:** 2023-05-12

**Authors:** Rajendra Upadhya, Woei C. Lam, Camaron R. Hole, Joseph G. Vasselli, Jennifer K. Lodge

**Affiliations:** ^1^ Department of Molecular Microbiology, Washington University School of Medicine, St. Louis, MO, United States; ^2^ Department of Molecular Genetics and Microbiology, Duke University School of Medicine, Durham, NC, United States

**Keywords:** *Cryptococcus*, cell wall, YNB medium, fungal virulence, chitosan, fungal PAMPs, chitin deacetylase

## Abstract

**Introduction:**

*Cryptococcus neoformans* is a basidiomycete fungus that can cause meningoencephalitis, especially in immunocompromised patients. Cryptococcus grows in many different media, although little attention has been paid to the role of growth conditions on the cryptococcal cell wall or on virulence.

**Objective:**

The purpose of this study was to determine how different media influenced the amount of chitin and chitosan in the cell wall, which in turn impacted the cell wall architecture and host response.

**Methods:**

Yeast extract, peptone, and dextrose (YPD) and yeast nitrogen base (YNB) are two commonly used media for growing Cryptococcus before use in in vitro or in vivo experiments. As a result, *C. neoformans* was grown in either YPD or YNB, which were either left unbuffered or buffered to pH 7 with MOPS. These cells were then labeled with cell wall-specific fluorescent probes to determine the amounts of various cell wall components. In addition, these cells were employed in animal virulence studies using the murine inhalation model of infection.

**Results:**

We observed that the growth of wild-type *C. neoformans* KN99 significantly changes the pH of unbuffered media during growth. It raises the pH to 8.0 when grown in unbuffered YPD but lowers the pH to 2.0 when grown in unbuffered YNB (YNB-U). Importantly, the composition of the cell wall was substantially impacted by growth in different media. Cells grown in YNB-U exhibited a 90% reduction in chitosan, the deacetylated form of chitin, compared with cells grown in YPD. The decrease in pH and chitosan in the YNB-U-grown cells was associated with a significant increase in some pathogen-associated molecular patterns on the surface of cells compared with cells grown in YPD or YNB, pH 7. This altered cell wall architecture resulted in a significant reduction in virulence when tested using a murine model of infection. Furthermore, when heat-killed cells were used as the inoculum, KN99 cells grown in YNB-U caused an aberrant hyper-inflammatory response in the lungs, resulting in rapid animal death. In contrast, heat-killed KN99 cells grown in YNB, pH 7, caused little to no inflammatory response in the host lung, but, when used as a vaccine, they conferred a robust protective response against a subsequent challenge infection with the virulent KN99 cells.

**Conclusion:**

These findings emphasize the importance of culture media and pH during growth in shaping the content and organization of the *C. neoformans* cell wall, as well as their impact on fungal virulence and the host response.

## Importance

Cryptococcosis, a potentially fatal infection caused by members of the *Cryptococcus neoformans* and *Cryptococcus gattii* species complex, is a major concern because of its unacceptably high incidence and mortality, particularly in resource-limited areas. Due to the limited availability of antifungals and the lack of an effective vaccine, there is an urgent need to discover effective treatments for this infection. The type of pathogen-associated molecular patterns on the fungal cell wall defines the nature of the host immune response during mammalian infection. We show that altering the culture media used to grow yeast cells results in significantly altered cell walls in wild-type KN99 cells. As a result, this medium-dependent cell wall modification has a significant effect on fungal virulence and pathogenicity in the mammalian host. The findings presented here will help define the fungus-specific components that are absolutely necessary for developing a safe and effective vaccine.

## Introduction

*Cryptococcus neoformans* and closely related species are basidiomycetes fungi that live in the environment, primarily in soil, pigeon guano, and certain tree species with a global distribution (Lin and Heitman, 2006; Barnett, 2010; Spickler, 2013). *C. neoformans* is a human pathogen that can infect the lungs and central nervous system, especially in patients with compromised immunity (mostly those with AIDS), eventually leading to meningoencephalitis, which is fatal if left untreated (Lin and Heitman, 2006; Park et al., 2009; Rajasingham et al., 2022). Despite the fact that it is a free-living budding yeast, it has the ability to switch between morphotypes under certain conditions. (Lin, 2009; Wang et al., 2012; Trevijano-Contador et al., 2016). *Cryptococcus* has been shown to survive in the environment inside soil amoeba (Steenbergen et al., 2001; Casadevall, 2012). Likewise, in the mammalian host it has the ability to subvert macrophage function and can divide and grow within the macrophages (Feldmesser et al., 2001; Gilbert et al., 2014). It spreads to multiple organs during mammalian infection, with a particular preference for the brain, and has the ability to proliferate in a variety of tissue environments, resulting in systemic infection (Strickland and Shi, 2021). This ability of *Cryptococcus* to adapt and grow in various external environments and host conditions demonstrates the unusual plasticity of its cell wall and the presence of efficient cellular mechanisms to combat different external stresses.

Many of the phenotypes discovered in *Cryptococcus* as part of its adaptive mechanisms to various environmental and host conditions have been identified as features associated with its virulence (Zaragoza, 2019; Zhao and Lin, 2021). Some of these include thermo-tolerance, polysaccharide capsule formation, chitosan production, Titan cell development, resistance to oxidative and nitrosative stress, and the expression of enzymes such as proteases, urease, and the anti-oxidant melanin (Missall et al., 2004a; Kronstad et al., 2011; Garcia-Rodas and Zaragoza, 2012). Specific culture conditions and growth parameters have been optimized in the laboratory to allow fungal cells to express specific virulent traits (Baker et al., 2007). For example, various media formulations have been optimized for the induction of polysaccharide capsule *in vitro* in order to replicate the phenotype observed during mammalian infection (Zaragoza and Casadevall, 2004). Likewise, to generate Titan cells *in vitro*, multiple culture media, growth protocols, and environmental and host-related cues have been identified (Dambuza et al., 2018; Hommel et al., 2018; Trevijano-Contador et al., 2018). Yeast extract, Peptone and Dextrose (YPD), Yeast Nitrogen Base (YNB), Roswell Park Memorial Institute (RPMI-1640) or Dulbecco's Modified Eagle Medium (DMEM), both containing 10% Fetal Bovine Serum (FBS) in the presence of 5% CO_2_ at 37°C are some of the commonly used media types routinely employed either to grow or to induce virulence traits. The development and optimization of *in vitro* laboratory conditions to replicate an *in vivo* virulence-related trait displayed by the fungus during mammalian infection has been critical in establishing a link between a specific trait and a gene in fungal pathogenesis.

The cell wall is a sophisticated organelle with a dynamic and flexible structure. It is essential for fungal survival and is the primary organelle through which fungal cells sense their surroundings. Importantly, the major fungal pathogen-associated molecular patterns (PAMPs), such as glucans, chitin, and mannoproteins, are integral components of the cell wall. Major variations in the content and organization of the cell wall have been reported in *Candida* and *Cryptococcus* cells grown in YPD and those grown under other laboratory culture conditions (Farkas et al., 2009; O’Meara et al., 2013; Weerasekera et al., 2016; Sherrington et al., 2017; Esher et al., 2018). One *C. neoformans* serotype A clinical isolate was reported to cause acidification of the medium when cultured in YNB, resulting in autolysis and secondary cell wall formation (Farkas et al., 2009). Titanization of *C. neoformans* induced either by growing the fungus in YNB medium containing 10% serum or by exposing the YNB-grown fungal cells to 10% HI-FCS (heat-inactivated fetal calf serum) was associated with a substantial alteration in the exposure of PAMPs on the cell surface (Dambuza et al., 2018; Hommel et al., 2018; Trevijano-Contador et al., 2018).

Chitin is one of the PAMPs found in the cell wall of *Cryptococcus*. Unlike many other yeasts, *Cryptococcus* converts most of its chitin into its deacetylated form, chitosan. Chitosan is required for cryptococcal virulence, and chitosan deficiency alters the host response, inducing a protective Th1-biased immune response (Baker et al., 2011; Upadhya et al., 2018; Lam et al., 2019; Hole et al., 2020). The activities of chitin synthase-3 (Chs3p) and chitin synthase regulator-2 (Csr2p), in conjunction with at least one of the chitin deacetylases (Cdas), are required for the synthesis of chitosan in both *C. neoformans* and *C. gattii* (Baker et al., 2007; Lam et al., 2019). The role of a specific Cda in chitosan production is determined by the external environment in which fungal cells grow (Upadhya et al., 2018; Lam et al., 2019). When cells are grown in YPD medium, all three Cdas are functionally redundant. The *CDA1* gene in *C. neoformans*, on the other hand, plays an important role in the production of chitosan during mammalian infection (Upadhya et al., 2018). However, in *C. gattii*, it is *CDA3* gene that is responsible for the production of most chitosan during mammalian infection. The reliance on either Cda1p or Cda3p for chitosan production in the host lung can be replicated in both cases if fungal cells are grown in a host-mimicking conditions, that is, on RPMI-1640 medium containing 10% FBS in the presence of 5% CO_2_ (Upadhya et al., 2018; Lam et al., 2019). These findings suggest that chitosan biosynthesis is highly dependent on the culture conditions used for fungal growth. Understanding the regulation of chitosan biosynthesis is therefore critical for unraveling the mechanisms of fungal virulence. Furthermore, the cryptococcal chitosan synthetic machinery will be a promising therapeutic target.

In this study, we investigated how the culture medium affects the amount of chitosan in the cell wall as well as the consequences of decreased chitosan in the cell wall (i.e., modified cell wall organization, increased exposure of PAMPs on the surface, and altered host response and virulence) of wild-type KN99 cells. Previously, we had grown *Cryptococcus* cells in unbuffered YPD before inoculating them into mice for virulence studies (Missall et al., 2004b; Gilbert et al., 2010; Baker et al., 2011; Upadhya et al., 2016; Upadhya et al., 2017; Upadhya et al., 2018). We chose to grow the cells in YNB for this study because it is a defined nutrient medium with previously demonstrated effects on cell size and call wall integrity (Farkas et al., 2009; Dambuza et al., 2018; Hommel et al., 2018; Trevijano-Contador et al., 2018). Here we show that, when yeast cells are grown in YPD or YNB without any buffering agents (YPD-U or YNB-U), the pH of the culture medium changes significantly and that the direction of the change is dependent on the medium. When cells are grown in YPD-U, the medium becomes slightly basic, whereas cells grown in YNB-U produce a very acidic medium. Importantly, the cells’ chitosan content varies significantly between the two media, with YNB-U cells having very little chitosan. The dramatic acidification is avoided by buffering YNB with 3-morpholinopropane-1-sulfonic acid (MOPS) to a pH of 7 (YNB, pH 7). We show that PAMP exposure in cells grown in YNB, pH 7, and host response to such cells, differ significantly from those seen in cells grown in YNB-U. Cells grown in YNB-U elicit a strong damaging, inflammatory response, demonstrating that the growth conditions prior to infection can influence how the host responds to *Cryptococcus* infection. Furthermore, chitosan synthesis was inhibited in YNB-U-grown cells compared with cells grown in YNB, pH 7. Chitosan deficiency in cells grown in YNB-U was associated with significantly reduced virulence when tested in a murine model using intranasal infection. We discovered that, when heat-killed (HK) cells were used as an inoculum, increased exposure of PAMPs on the surface of the YNB-U-grown cells caused an exuberant inflammatory reaction in the lungs in an inoculum dose-dependent manner, leading to early disease and death of the infected animals at a higher inoculum. When compared with cells grown in YPD or YNB-U, KN99 cells grown in YNB, pH 7, had intermediate levels of chitosan in the cell wall. This amount of chitosan in the cell wall was associated with an efficient shielding of PAMPs on the cell surface. Consistent with this, when HK cells grown in YNB, pH 7, were used for infection, they resulted in a muted inflammatory response in the lung. Even with this muted inflammatory response, mice inoculated with a HK inoculum of KN99 cells grown in YNB, pH 7, at a dose equivalent to 10^7^–10^8^ colony-forming units (CFU) induced a robust protective response to a subsequent challenge infection with KN99 cells. In CBA/J strains, a single dose of vaccination was sufficient to elicit a robust protective response, whereas C57BL/6 strains of mice required a booster dose of vaccination to elicit a strong protective immunity.

## Results

### The amount of chitosan in the cell wall depends on the type of medium used to cultivate *C neoformans*

**Figure 1:**
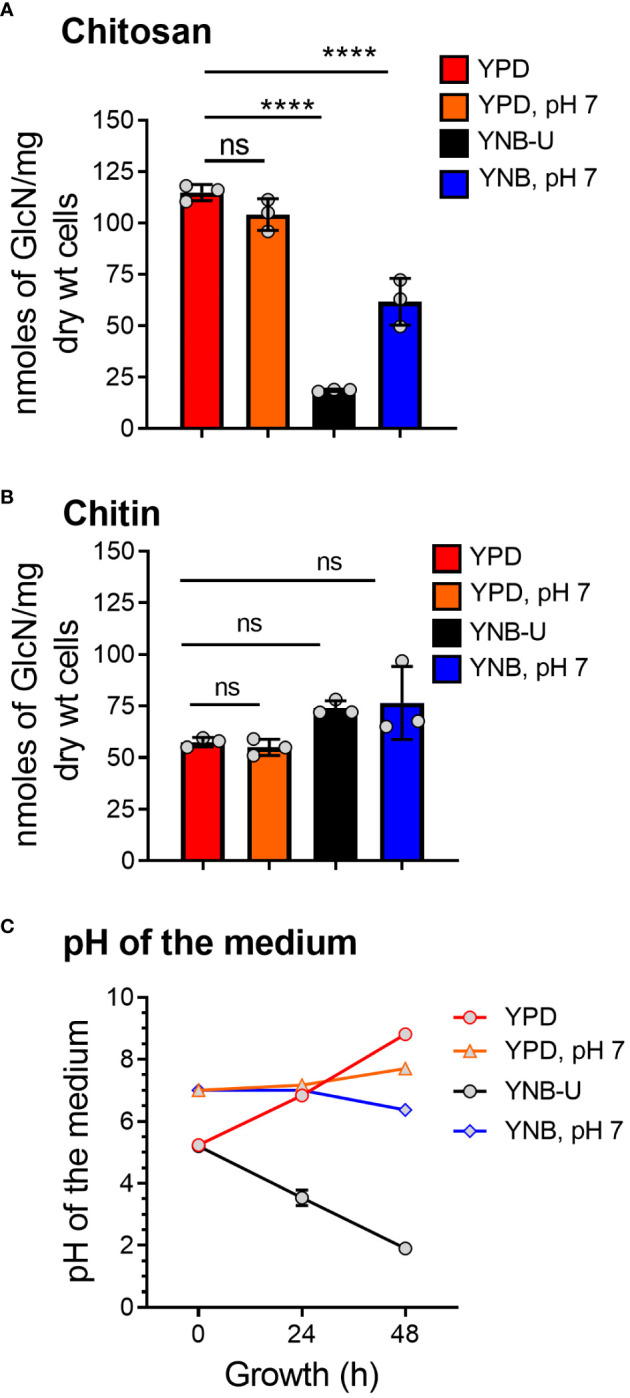
The amount of chitosan in the *C*. *neoformans* cell wall is greatly influenced by the type of growth medium used. **(A)** The amount of chitosan was measured using the MBTH method after wild-type KN99 cells were grown in the appropriate medium for 48 h at 30°C. **(B)** The samples as shown in A were subjected to total chitin measurement by the MBTH method. **(C)** The pH of the spent medium was measured at 0, 24, and 48 h during the growth of KN99 cells in the respective medium at 30°C. Data represent the averages of three biological experiments. Significant differences between the groups were compared using one-way ANOVA followed by Dunnett’s multiple-comparison test. ****p < 0.0001 when comparing the amount of chitin in cells grown in YPD compared with that in cells grown in other media. ns, not significant.

We previously demonstrated that chitin, in the form of deacetylated chitosan, is required for cell wall integrity and fungal virulence in *C. neoformans* (Baker et al., 2007; Baker et al., 2011; Upadhya et al., 2018). As a result, we decided to see if the culture medium used to grow *C. neoformans* had any effect on chitosan biosynthesis. When the cells were grown in YNB-U medium, we discovered that their ability to synthesize chitosan was severely hampered. As shown in **Figure 1A**, the amount of chitosan present in the cell wall of yeast cells grown in YNB-U medium was 6.4 times less than that of yeast cells grown in YPD medium, as measured by the MBTH (3-methyl-2-benzothiazolinone hydrazone hydrochloride) method. However, if the pH of the YNB medium was pre-adjusted to a neutral pH of 7 with 50 mM MOPS (YNB, pH 7), the amount of chitosan in these cells decreased two-fold compared with YPD-grown cells (**Figure 1A**). Compared with cells grown in unbuffered YPD medium, adjusting the initial pH of YPD medium with MOPS to a pH of 7 had no effect on chitosan synthesis during growth (**Figure 1A**; comparing YPD with YPD, pH 7). However, the composition of the growth medium, in contrast to its effect on chitosan, had no effect on the levels of its precursor, chitin. We measured total chitin using the MBTH method and discovered a comparable amount of chitin after 48 h of growth in either medium, with or without MOPS (**Figure 1B**). We chose to buffer the medium to a physiological pH of 7 because *C. neoformans* growth was associated with a significant change in the pH of the growth medium, with the direction of effect varying depending on the growth medium used. When yeast cells were grown in YPD, their growth was accompanied by a shift in the pH of the medium from 5.2 to 8.0 in 48 h (**Figure 1C**). Cell growth in YNB medium, on the other hand, resulted in a dramatic decrease in pH from 5.2 to 2.0 after 48 h (**Figure 1C**). This change in pH due to growth was prevented by pre-adjusting the pH of the medium to a pH of 7.0 by supplementing with 50 mM MOPS before inoculation (**Figure 1C**; YPD, pH 7, and YNB, pH 7). These findings imply that the type of growth medium used to cultivate *C. neoformans* has a specific impact on chitosan production, which is mediated by both nutrient limitation (comparing chitosan content in YPD grown cells to YNB) and pH changes during growth.

### Chitosan deficiency in YNB-U cells is associated with altered morphology and compromised cell wall integrity

**Figure 2:**
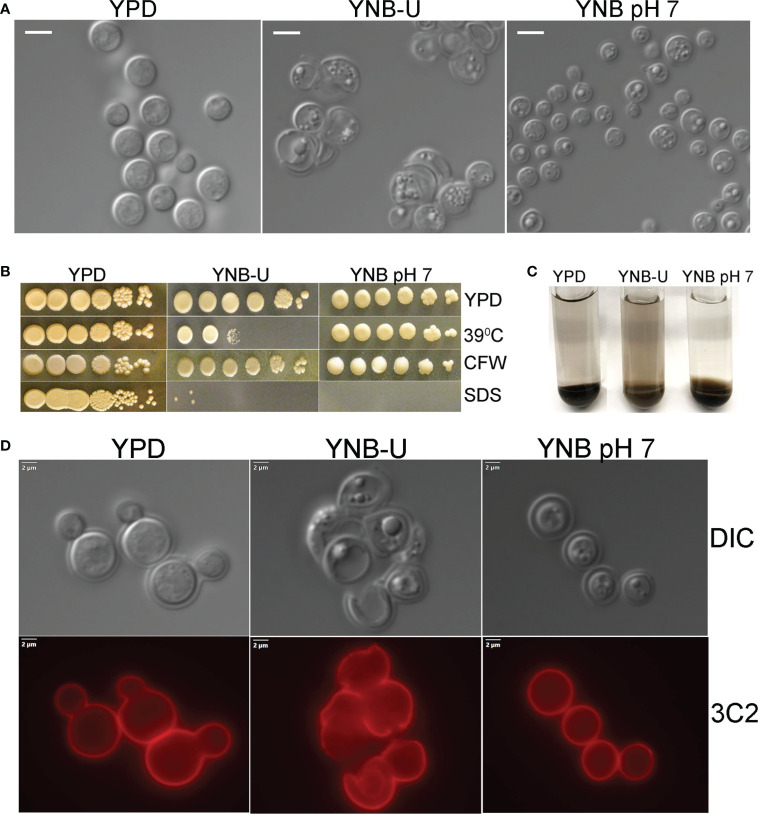
Cell wall integrity is impaired in YNB-U-grown cells, but there is no major defect in the capsule. **(A)** Differential interference contrast (DIC) images of cells grown in the indicated medium for 48 h The growth of KN99 cells in YNB-U medium resulted in an altered cell wall morphology. **(B)** Cells grown in either YNB-U or YNB, pH 7, show a defective cell wall. Serial dilutions of the cells grown for 48 h in the respective medium were spotted onto YPD containing specified cell wall stressors or at the indicated temperatures. CFW, calcofluor white; SDS, sodium dodecyl sulfate. **(C)** Assessment of leaky melanin phenotype. Cells grown for 48 h in the indicated medium were collected, washed in 1 × PBS, and resuspended to identical optical densities in glucose-free asparagine medium containing L-DOPA, and allowed to shake (300 rpm) at 30°C. Cells were centrifuged to visualize the secreted pigment and the color of the pellets. The presence of the pigment in the supernatant indicates a “leaky melanin” phenotype. **(D)** Representative immunofluorescence micrographs of KN99 cells after growth in the specified medium for 48 (h) Anti-GXM monoclonal antibody 3C2 and a goat anti-mouse IgG conjugated to Alexa Fluor^™^ Plus 555 were used to stain and visualize the capsule.

We observed that the inability of *C. neoformans* to synthesize chitosan during growth in the synthetic YNB-U medium resulted in altered cell morphology, with budding defects and often enlarged cells, as shown in **Figure 2A**. This abnormal morphology of the cells was prevented by buffering the pH of the medium to 7.0 with MOPS. Previously, we have shown that chitosan-deficient mutants of *C. neoformans* are sensitive to a variety of cell wall-perturbing agents (Baker et al., 2007). Therefore, we hypothesized that the lower levels of chitosan in the cell wall of yeast grown in YNB-U would impact the integrity of the cell wall. As shown in **Figure 2B**, cells grown in YNB-U were found to be sensitive to a higher temperature of 39°C. The cells grown in YNB medium displayed higher sensitivity to sodium dodecyl sulfate (SDS), regardless of whether the medium was buffered or not (**Figure 2B**). We have previously demonstrated that *C. neoformans* mutants lacking chitosan have a leaky melanin phenotype (Baker et al., 2007). Therefore, we wanted to see if YNB-U growth-induced chitosan deficiency affects melanin retention in the cell wall. Cells grown in YNB-U displayed a leaky melanin phenotype as shown in **Figure 2C** similar to what we had seen in chitosan-deficient mutants (Baker et al., 2007). These findings suggest that chitosan deficiency induced in wild-type KN99 cells by growth in YNB-U medium causes cell wall alterations that are similar to the cell wall remodeling induced by the deficiency of chitosan resulting from gene deletions.

The next step was to determine whether the amount of capsule surrounding the cells changed as they grew in different medium. As shown in **Figure S1A**, when *C. neoformans* was grown in any of the three types of growth medium, the capsule was not hyper-induced. We stained the cells with capsule-specific antibodies to ensure that the capsule was still being produced and was still attached to the cell. These capsule-bound antibodies were visualized using a fluorescent microscope after being labeled with fluorescently tagged secondary antibodies. Capsule labeling of cells cultured in three different types of medium for 48 h with either 3C2 (**Figure 2D**) or 18B7 antibodies (**Figure S1B**) revealed the absence of any deficiency in capsule synthesis or its attachment to the cell wall.

### The type of medium used for *C. neoformans* growth determines the nature of the PAMPs exposed on the cell surface

**Figure 3:**
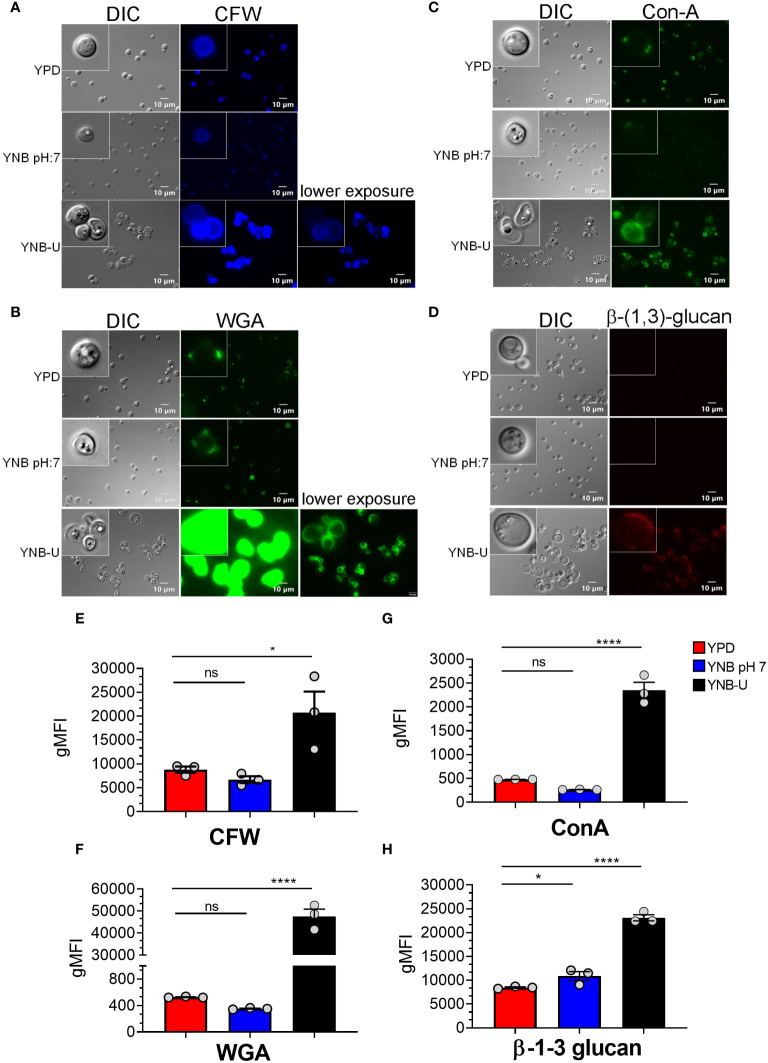
Pathogen associated molecular pattern (PAMPs) are abnormally exposed on the cell surface of wild-type KN99 cells grown in YNB-U, whereas growth in YNB, pH 7, induces substantial masking at the surface. KN99 cells were grown for 48 h in the indicated medium. Paraformaldehyde-fixed cells were stained with CFW for chitin **(A)**, FITC-conjugated WGA for chito-oligomers **(B)**, FITC-conjugated ConA for mannoproteins **(C)**, and β-1,3-glucan-specific antibody **(D)**. Representative images of labeled cells captured using fluorescent microscopy **(A–D)**. Flow cytometry was used to calculate the geometric mean fluorescence intensity (gMFI) of stains associated with yeast cells **(E–H)**. *p < 0.003 and ****p < 0.0001 as determined by one-way ANOVA after Dunnett’s multiple comparisons test with cells grown in YPD as control group. ns, not significant. Three biological replicates were analyzed for each condition. DIC, differential interference contrast; WGA, wheat germ agglutinin.

Molecules that localize to the fungal cell wall are important for sensing the external environment and assisting yeast cells by activating intracellular signaling pathways that aid in the cells’ adaptation and stress responses. As part of an adaptive mechanism, changes in the composition and/or reorganization of cell wall components will activate specific cellular signaling pathways, which then coordinate specific gene expression programs within the cell. The cell wall of *C. neoformans* is mainly composed of polysaccharides such as glucans (α-1,3-glucan, β-1,3-glucans, and β-1,6-glucans), chitin, chitosan, mannoproteins, and glycosylphosphatidylinositol (GPI)-anchored proteins. Various cell wall/membrane-specific fluorescent dyes or modified biomolecules have previously been used as tools to probe and distinguish specific cellular components from one another, to determine their specific levels, and to recognize their organization relative to one another (Okada and Ohya, 2016; Esher et al., 2018; Bloom et al., 2019; Lichius and Zeilinger, 2019; Bloom et al., 2021). As a result, we stained KN99 cells grown in the three different media with (1) Calcofluor White (CFW), to determine the amount of chitin; (2) fluorescein isothiocyanate (FITC)-conjugated wheat germ agglutinin (WGA), to specifically quantify surface exposed chito-oligomers; (3) FITC-conjugated concanavalin A (Con A), to determine the abundance of mannoproteins exposed on the surface; and (4) β-1,3-glucan-specific mouse monoclonal antibody to assess the degree of unmasking of β-1,3-glucans. Representative fluorescence microscopy images of the samples are shown in **Figures 3A–D**, while **Figures 3E–H** show the average fluorescence intensities of the labeled cells as determined by flow cytometric analyses.

All the PAMP-specific probes were more abundant in yeast cells grown in YNB-U medium than in yeast cells grown in YPD or YNB, pH 7; however, the binding patterns of each of the cell wall-specific probes to the cells grown in three different media were not substantially different (**Figures 3A–D**). We did observe a significant difference in the average fluorescence intensity between the three cell types using different probes. The mean fluorescence intensity due to CFW staining resulted in a modest (two- to three-fold) increase in CFW binding to YNB-U-grown cells when compared with cells grown in either YPD or YNB, pH 7 (**Figures 3A, E**). The intensity of fluorescence induced by FITC-conjugated WGA in cells grown in YNB-U was 91 and 135 times higher than in cells grown in YPD or YNB, pH 7, respectively (**Figures 3B, F**). Mannoprotein exposure was five times higher in cells grown in YNB-U medium than in cells grown in either YPD or YNB, pH 7 (**Figures 3C, G**). The monoclonal antibody specific for β-(1,3)-glucan could bind only to yeast cells grown in YNB-U, implying that the polymer β-(1,3)-glucan is specifically unmasked only in YNB-U cells (**Figures 3D, H**). These data suggest that the cell wall changes resulting from growth in YNB-U medium expose the cell wall chitin, chito-oligomers, and β-(1,3)-glucan.

### The nature of the culture medium has a significant effect on the cell wall organization of *C. neoformans*

**Figure 4:**
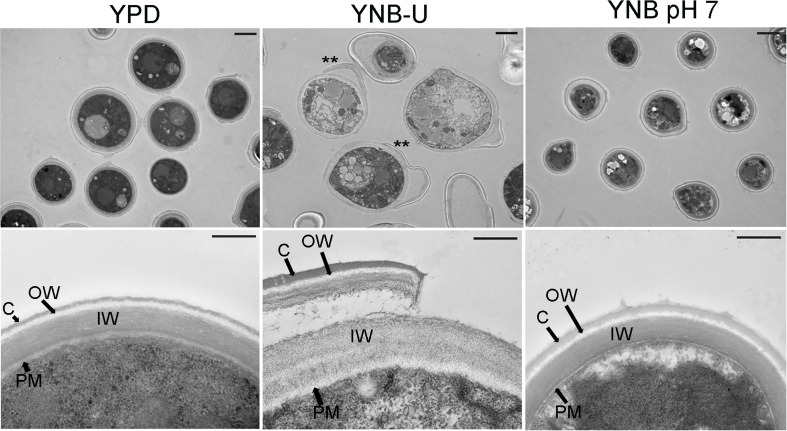
The cell wall architecture of *C. neoformans* is affected differently depending on the type of medium used for growth. Representative transmission electron micrographs of cells grown in three different media. KN99 cells were grown in the indicated medium for 48 h were collected and fixed. Fixed samples were further processed, embedded, sliced, and imaged by transmission electron micrography. Upper panel of images: scale bar, 2 µm; 3000× magnification. Lower panel of the images: scale bar, 0.5 µm; 25,000× magnification. C, capsule; OW, outer wall; IW, inner wall; PM, plasma membrane. Asterisks in the upper panel of the YNB-U indicate the dissociated part of the cell wall still attached to the parent cell.

To visualize the effect of the growth medium on the ultrastructure of the *C. neoformans* cell wall, we used transmission electron microscopy (TEM) to examine the cells grown in three separate media for 48 h. Cells grown in YPD and YNB at pH 7 exhibited a compact cell wall with a distinct inner wall (IW) sandwiched between a well-defined outer wall (OW) and the plasma membrane (PM) components (**Figure 4**). In both cases, the OW was surrounded by a capsular (C) layer, as previously described (Agustinho et al., 2018). The capsular layer appeared to be wider in cells grown in YNB, pH 7, than in cells grown in YPD (**Figure 4**). In contrast, the cells grown in YNB-U had a diffuse cell wall architecture with an electron-lucent plasma membrane. Furthermore, a portion of the IW, as well as the OW and capsule layer, was discovered to have been stripped away from the cells, revealing the IW fibrils. Lower-magnification images of the YNB-U-grown cells show the presence of these dissociating cell wall fragments, which are still adherent to the parent cell wall (**Figure 4**, upper panel of YNB-U with asterisk). The diffuse and eroded outer walls of YNB-U-grown cells are consistent with their compromised cell wall integrity and exposed PAMPs on the surface.

**Figure 5:**
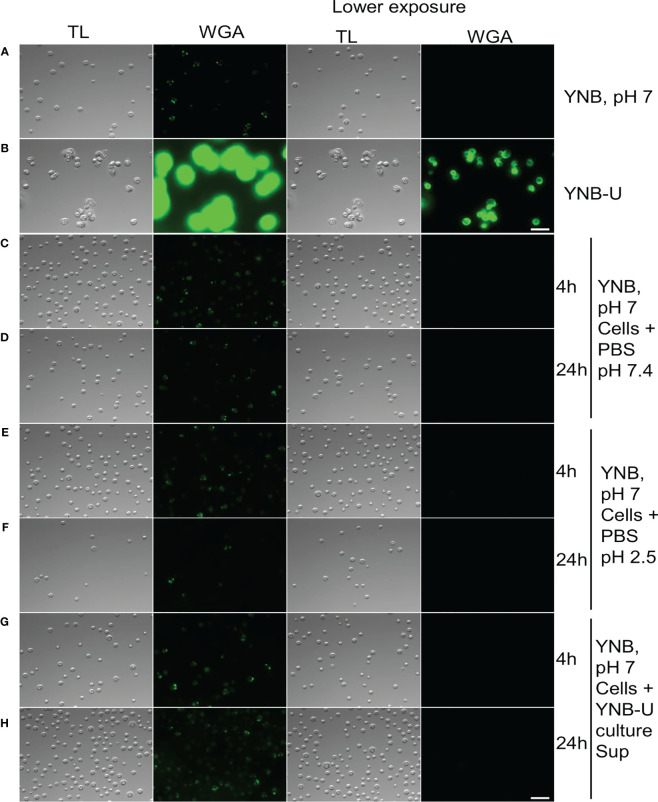
Cell wall remodeling was not induced by the acidity of the medium or any biological activity in the culture supernatant. KN99 cells were grown for 48 h at 30°C in either YNB, pH 7, or YNB-U medium. Cells were centrifuged and collected. YNB, pH 7-grown cells were used as test cells, while supernatant from YNB-U culture was used as a source of biological activity. Representative images of the WGA-labeled cells are shown. **(A)** Cells grown in YNB, pH 7. **(B)** Cells grown in YNB-U. Representative images of WGA-labeled YNB, pH 7-grown cells that were incubated either in PBS, pH 7.4, at 30°C for 4 h **(C)** or 24 h **(D)**; or in PBS, pH 2.5, for 4 h **(E)** or 24 h **(F)**; or in the supernatant of YNB-U culture for 4 h **(G)** or 24 h **(H)**. Because YNB-U-grown cells show intense staining at auto-focus settings, the same transmitted light images are shown with a lower exposure. All the samples are subjected to identical exposure times. Scale bar,10 µm. DIC, differential interference contrast; WGA, wheat germ agglutinin.

Several internal and external factors could contribute to cell wall damage when yeast is grown in YNB-U medium. Changes in the gene expression profiles of genes involved in cell wall synthesis or its maintenance, as well as direct effects on the function of critical proteins involved in cell wall biogenesis, are examples of internal factors. External factors include simple cell exposure to a medium with a highly acidic pH, or the activation of specific cell wall-hydrolyzing enzymes at acidic pH. We wanted to know if the cell wall damage observed in YNB-U-grown cells was caused by acidic pH or the action of specific hydrolyzing enzymes. To address this, we cultured KN99 cells in YNB, pH 7, and YNB-U media. The cells were centrifuged 48 h after they had grown. Cells grown in YNB at pH 7 served as the substrate, while the YNB-U culture supernatant was saved as a source of acid-active cell wall-hydrolyzing enzymes. Cells grown in YNB, pH 7, were washed with PBS and then incubated at 30°C in PBS-pH 7, PBS-pH 2.5, and YNB-U supernatant. At 4 h and 24 h after incubation, cells were collected and labeled with WGA as described above. Incubating cells in PBS-pH 2.5 for either 4 h or 24 h (**Figures 5A–F**) or in YNB-U culture supernatant for either 4 h or 24 h (**Figures 5G, H**) did not result in increased WGA binding to the surface of YNB, pH 7-grown cells. These findings show that simply exposing *C. neoformans* cells to an acidic pH does not result in significant cell wall damage. Furthermore, the absence of increased WGA binding after incubating yeast cells in YNB-U supernatant rules out the presence of any secreted cell wall-hydrolyzing enzymes in the YNB-U culture supernatant. It will be interesting to see, in future studies, if acidic pH-activated cell wall-bound enzymes are involved in causing the cell wall damage observed in cells grown in YNB-U medium.

### Growth of KN99 cells in YNB-U significantly impacts its virulence in a murine infection model

**Figure 6:**
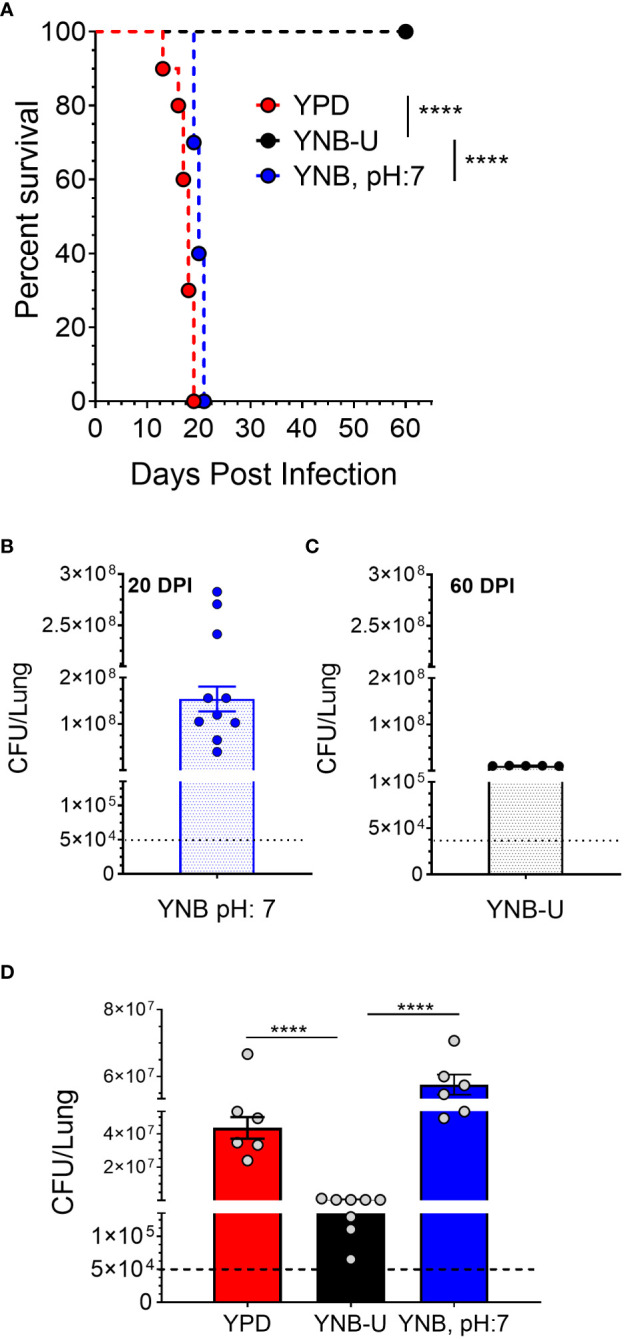
In a murine infection model, wild-type KN99 cells grown in YNB-U showed significantly reduced virulence. **(A)** Female CBA/J mice (4–6 weeks old) were intranasally infected with 5 × 10^4^ CFU of live KN99 cells grown in three separate culture media. Survival of the animals was recorded as mortality of mice for 60 days post infection (DPI). Mice that lost 20% of their body weight were deemed sick and sacrificed. Data are representative of two independent experiments with five animals for each culture. Virulence was determined using Mantel-Cox curve comparison with statistical significance determined by the log rank test ****p < 0.0001. **(B)** Fungal burden in the lungs of mice infected with cells grown in YNB, pH 7, at the end point of the survival experiment (20 DPI). **(C)** Fungal burden in the lungs of mice infected with the cells grown in YNB-U at the end point of the survival experiment (60 DPI). **(D)** Fungal load in the lungs of the mice infected with wild-type KN99 cells grown in three different media at 14 DPI. Data are the average of two experiments with three or four animals per group. ****p < 0.0001 as determined by one-way ANOVA after Tukey’s multiple comparisons test. The dashed line indicates the CFU of the inoculum.

The total amount of PAMPs on the surface of yeast cells, as well as the extent to which they are exposed, has been shown to influence the nature of the host immune response, thereby affecting fungal virulence (O’Meara et al., 2013; Campuzano and Wormley, 2018; Esher et al., 2018). We previously demonstrated that mutants of *C. neoformans* or *C. gattii* R265 that are unable to synthesize chitosan were avirulent in a murine infection model (Baker et al., 2011; Upadhya et al., 2018; Lam et al., 2019; Hole et al., 2020). As a result, we hypothesized that the small amount of chitosan present in YNB-U-grown cells might affect their virulence. We grew KN99 cells in three different media (YPD, YUB-U, and YNB, pH7), and a live inoculum corresponding to 50,000 CFU was used for intranasal inoculation of CBA/J mice. The virulence levels were determined using the procedures outlined in the Materials and methods section. Cells grown in YNB-U were avirulent for up to 60 days post infection (DPI), as shown in **Figure 6A**. Cells grown in YPD or YNB, pH 7, had comparable virulence, with median survival times of 18 and 20 days, respectively (**Figure 6A**). Surprisingly, mice infected with live KN99 cells grown in YNB-U survived the experiment and showed no clinical signs of cryptococcosis. However, when we examined the lungs of the mice for fungal burden at the end of the survival experiment (60 DPI), we discovered an average of 1 × 10^7^ CFU in the lungs (**Figure 6C**). In two biological experiments, one of 10 animals showed signs of *Cryptococcus* infection, including a pronounced hunchback and unbalanced movement. We discovered CFU in this mouse’s brain, indicating that the fungal cells eventually made their way to the brain, causing the classic symptoms of cryptococcal infection. The remaining nine animals appeared to be in good health, with no fungal cells in their brains. By contrast, growing the cells in YNB, pH 7, restored fungal virulence, with fungal burden in the lungs reaching an average CFU of 1.54 × 10^8^ at about day 20 post infection (**Figure 6B**).

The observed differences in virulence could reflect a difference in the initial growth rate of inoculum in the host lung between cells grown in three different media. We inoculated mice with 50,000 CFU of live KN99 cells grown in three different types of medium and measured the CFU in the animal lungs at day 14 post infection. As shown in **Figure 6D**, KN99 cells grown in YPD and YNB, pH 7, showed similar growth, with an average increase in CFU of three logs of (800- to 1,200 fold) compared with their inoculum levels. In contrast, mice inoculated with KN99 cells grown in YNB-U had an average of 476,500 CFU, which is roughly 10-fold higher than the initial inoculum levels. CFU in the lungs of mice infected with cells grown in YNB-U were 91 and 126 times lower, respectively, than CFU in the lungs of mice infected with YPD- and YNB, pH 7-grown cells (**Figure 6D**). These findings imply either that cells grown in YNB-U proliferate slowly in the murine lung environment or that the altered cell wall architecture caused some of the inoculated cells to be cleared from the host.

### Mice die quickly after being inoculated with HK wild-type KN99 cells grown in YNB-U

**Figure 7:**
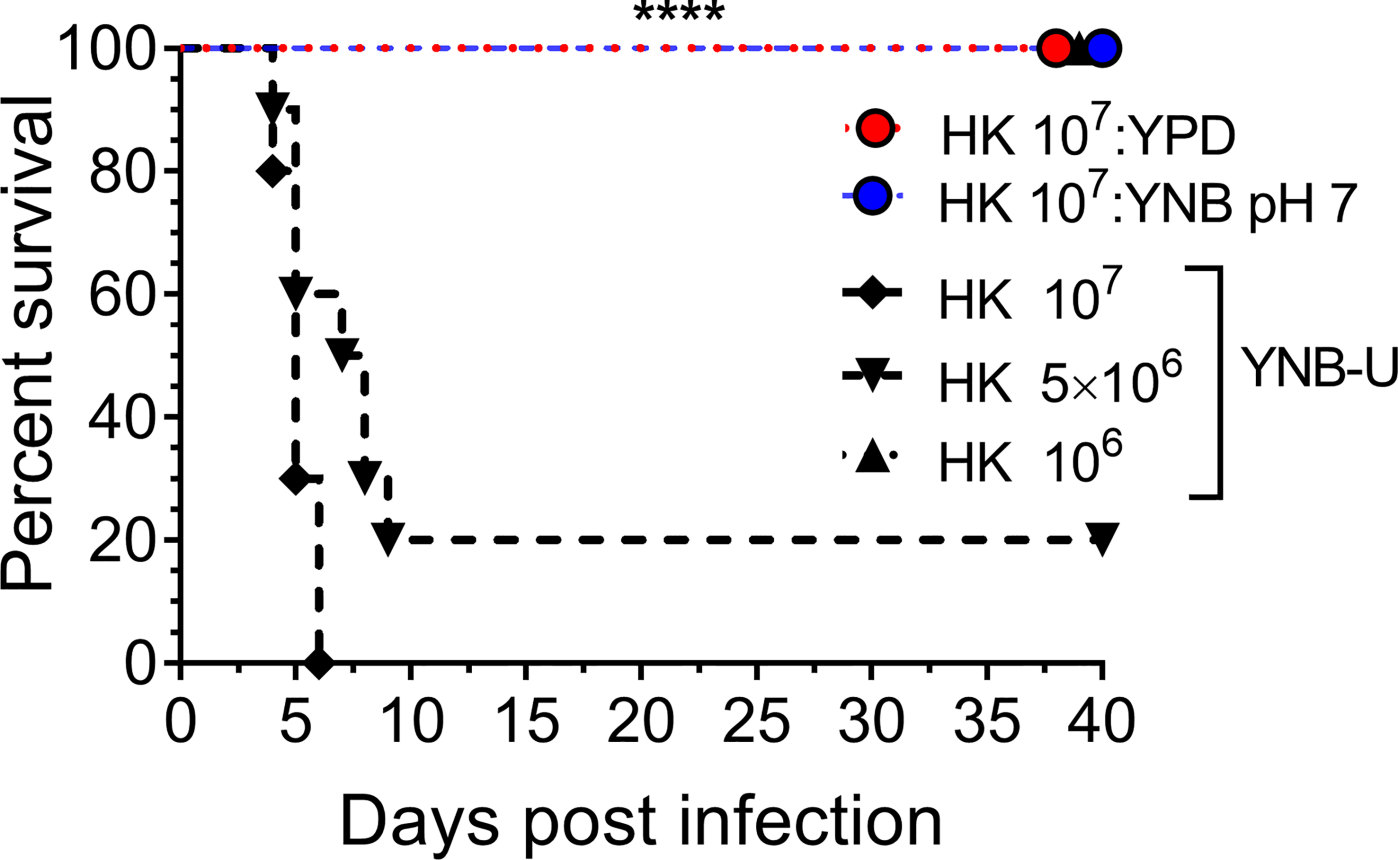
YNB-U-grown KN99 cells used for infection after heat killing cause inoculum dose-dependent mouse mortality. Female CBA/J mice (4–6 weeks old) were given the indicated doses of heat-killed KN99 cells grown in the specified culture medium. The animals’ survival was measured by the mortality of mice 40 days after intranasal infection. Mice that lost 20% of their body weight or showed signs of morbidity at the time of inoculation were considered ill and sacrificed. Except for the data for the group infected with 10^6^ HK cells of YNB-U, which is from one experiment with five mice, each group’s data are the sum of two experiments with five mice. The Mantel–Cox curve comparison was used to assess survival, with the log-rank test used to determine statistical significance (****p < 0.0001).

We previously demonstrated that using HK cells of chitosan-deficient *C. neoformans* derived from the deletion of all three CDAs (*cda1Δcda2Δcda3Δ*) as a vaccine, at a dose of 10^7^ CFU, induced protective immunity (Upadhya et al., 2016). In contrast, we also observed that inoculating mice with an identical dose of HK *chs3*Δ, a different chitosan-deficient mutant, resulted in the animals’ death due to a neutrophil-mediated hyper-inflammatory response in the lungs (Hole et al., 2020). Therefore, we wanted to see if the physiological lack of chitosan caused by the growth medium had a similar effect on the host. To that end, we inoculated CBA/J mice with three separate doses of HK KN99 cells grown in YNB-U. We found that incubating the yeast cells at 70°C for 15 min was sufficient to kill them, as no colonies emerged when we plated these HK cells on a YPD plate. Inoculating mice with a dose equivalent to 10^7^ CFU of HK cells resulted in the animals dying at 6 DPI, as shown in **Figure 7**. The number of survivors increased when the inoculum dose was reduced to the equivalent of 5 × 10^6^ CFU, compared with the 10^7^ CFU dose, whereas a dose equivalent to 10^6^ CFU caused no mortality in the mice. While HK YPD and YNB, pH 7-grown cells were safe at a dose of 10^7^ (**Figure 7**), YNB-U-grown cells required a 10-fold lower dose to prevent animal death. As a result, we discovered that HK KN99 cells grown in YNB-U caused dose-dependent mouse mortality when administered intranasally.

### Wild-type KN99 cells grown in YNB-U cause a hyper-inflammatory response in the murine lung

The death of the animals following infection with HK KN99 cells grown in YNB-U (**Figure 6A**) suggests that the animals are dying not as a result of fungal growth in the lungs, but rather as a result of host-mediated mortality. To test this, we inoculated mice with an HK inoculum containing 10^7^ CFU of KN99 cells grown in three different media. At day 3 post infection, lungs were processed for histology. Histological examination of the infected lungs revealed significant infiltration of immune cells in all three experimental mouse groups (**Figure S2**). Mice infected with KN99 cells grown on YNB-U medium exhibited greater infiltration of immune cells in the airways and interstitial pulmonary tissue (**Figure S2**). Furthermore, YNB-U-infected mice lungs had a broken alveolar wall with air spaces filled with granular fluid, an indication of pulmonary edema. The animal mortality observed in the survival experiment (**Figure 6A**) is consistent with this type of lung damage. Despite the presence of immune cells in the lungs of mice infected with HK YPD- or YNB, pH 7-grown cells, the fact that these mice survived, as shown in **Figure 6A**, suggests that their lungs were not irreversibly damaged.

**Figure 8:**
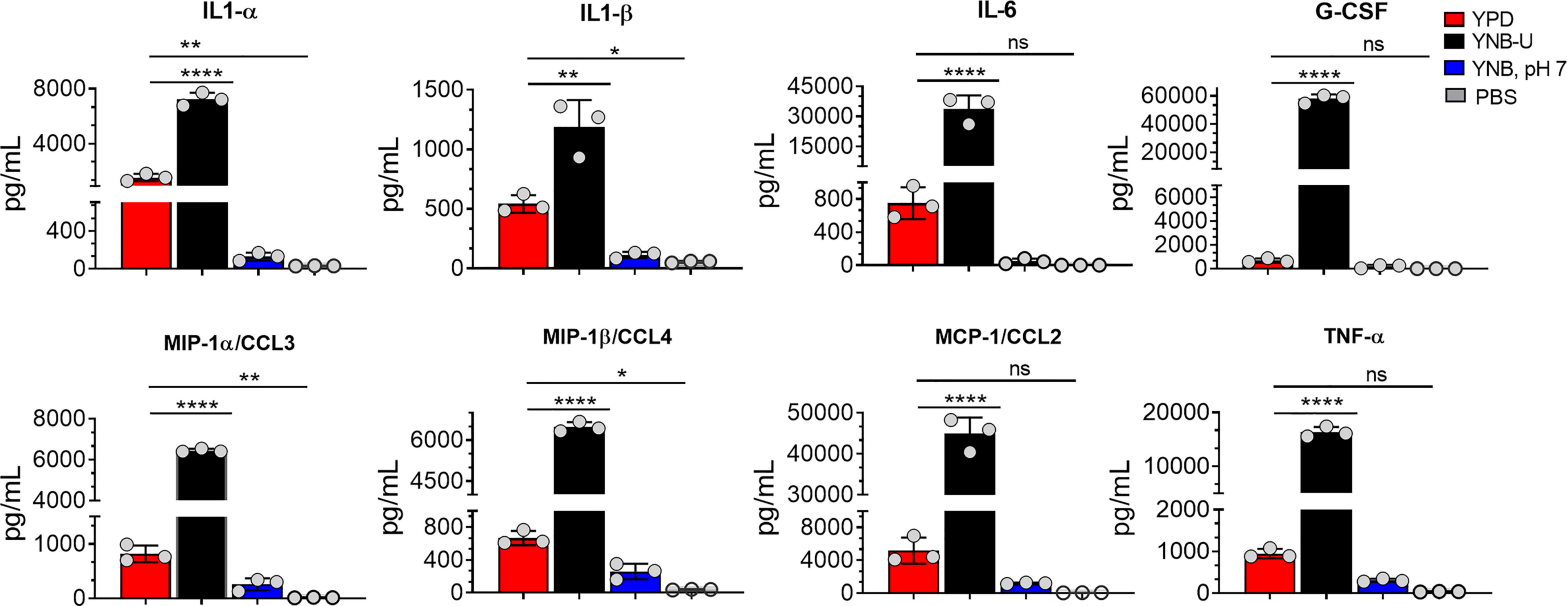
Cells grown in YNB-U induce a dramatic hyper-inflammatory response in the lung. Absolute levels of cytokines and chemokines in the lungs that showed a significant difference between any one of the experimental groups of mice on day 3 post-infection. CBA/J mice were inoculated with an inoculum dose corresponding to 10^7^ CFU of a HK preparation of KN99 cells grown in YPD, YNB-U, or YNB, pH 7, for 48 h. Lung homogenates were prepared and the cytokine/chemokine responses were determined using the Bio-Plex protein array system (Bio-Rad, USA). Data are representative of a minimum of two experiments with three animals each. All data are presented as mean values ± standard errors of the means (SEM). ns, not significant; ****p < 0.0001, **p < 0.005, and *p < 0.02 as determined by one-way ANOVA with Dunnett’s multiple comparisons test.

Since we observed significant infiltration of immune cells to the infected lungs (**Figure S2**), and given that mice infected with KN99 cells grown in YNB-U survived for only 5 DPI (**Figure 6A**; HK 10^7^ YNB-U), we decided to determine the cytokine/chemokine profile of the infected lungs. This was accomplished by inoculating the mice with a dose equal to 10^7^ CFU of HK KN99 preparations as described above. On days 3, 5, and 7 post infection, we prepared homogenates of the lungs of each group of mice. Only the cytokines/chemokines whose concentration in the lung differed significantly between any of the experimental groups at day 3 post infection and at an inoculum dose equivalent to 10^7^ CFU were compared and are shown in **Figure 8**. The remaining data are presented in **Figure S3**. At day 3 post infection, the cells grown in YNB-U induced a massive production of various cytokines and chemokines in the lung (**Figure 8**). In cells grown in YNB, pH 7, induction of any of the inflammatory molecules studied was minimal to non-existent (**Figure 8**). Despite the fact that cells grown in YPD produce more interleukin (IL) 1α, IL1-β, chemokine ligand 3 (CCL3), and CCL4 than cells grown in YNB, pH 7, the levels produced by YPD-grown cells are significantly lower than those produced by YNB-U-grown cells. When the cytokine levels in lungs inoculated with YNB-U-grown cells were compared with those in lungs inoculated with YPD-grown cells, IL-6 and granulocyte colony-stimulating factor (G-CSF) levels were found to be several times higher (45- and 85-fold, respectively; **Figure 8**). In the lungs of mice infected with YNB-U-grown cells, the CCL family of chemokines (CCL2, CCL3, and CCL4) were all significantly up-regulated in comparison with YPD/YNB, pH 7-grown cells. CCL2 has been demonstrated to be an effective neutrophil chemoattractant in the lung, contributing to acute inflammation (Upadhya et al., 2016). When YNB-U-infected lungs were compared with YPD-infected lungs, TNF-α levels were found to be 17 times higher in the YNB-U-infected lungs.

To determine if the inflammatory response was dose dependent, we compared a 5 × 10^6^ CFU inoculum dose for the cells grown in YNB-U to the 10^7^ inoculum dose. On days 3, 5, and 7 post infection, we prepared homogenates of each group of mice’s lungs. The degree of activation of certain cytokines/chemokines differed significantly between the experimental groups of mice (**Figure S3**). The amount of cytokine produced in YNB-U-grown cells was found to be proportional to the dose of inoculum, with a 10^7^ CFU dose activating more cytokines in the lungs than a 5 × 10^6^ CFU dose (**Figure S3**). The amount of cytokine/chemokines produced was highest on day 3 after infection, regardless of inoculum dose or type, before decreasing on days 5 and 7 in the mice infected with cells grown in either YPD or YNB, pH 7 (**Figure S3**).

### Heat-killed KN99 cells grown in YNB, pH 7, induce a strong protective response against *C. neoformans* infection in both CBA/J and C57BL/6 mouse strains

**Figure 9:**
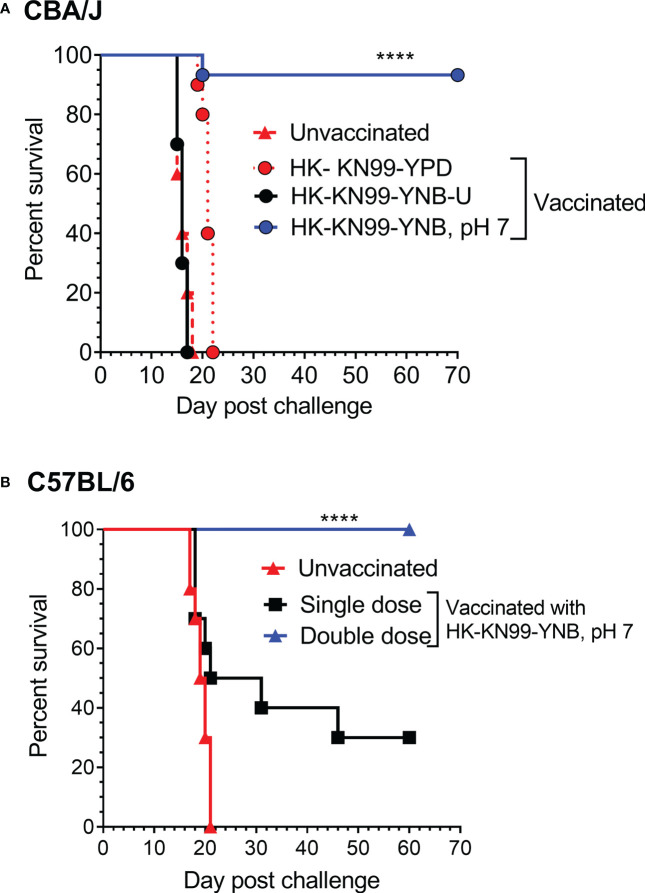
Growth medium-induced cell wall modifications render wild-type KN99 cells an effective vaccine against *C*. *neoformans* infection. **(A)** CBA/J mice were vaccinated through inhalation with the HK preparation of KN99 cells (10^7^ CFU) grown in the indicated culture medium. Animals were left for 40 days to allow the infection to resolve. Subsequently, all groups of mice were challenged with 5 × 10^4^ CFU of live KN99 cells. The death rate of mice was recorded as a measure of virulence. Mice that had lost 20% of their starting body weight were deemed moribund and sacrificed. The percentage of mice that survived was plotted against the DPI. Data are cumulative of one experiment with five mice for PBS, YPD, and YNB-U (10^6^ CFU dose) and three experiments with five mice each for KN99, YNB pH 7. **(B)** C57BL/6 mice were vaccinated with a dose equivalent of 10^8^ CFU of a HK preparation of KN99 cells grown in YNB, pH 7. At –40 days, the single-dose group received a single dose of vaccine, while the double-dose group received a booster dose at –28 days. The animals were quarantined for 40 days to allow the infection to resolve. As stated above, challenge infection and survival were tracked. The data are a combination of two experiments with five animals in each group. The Mantel–Cox curve comparison was used to measure survival, with the log-rank test used to determine statistical significance (****p < 0.0001).

We previously reported not only that *C. neoformans* chitosan-deficient mutants elicit a robust pro-inflammatory cytokine/chemokine response in the lungs, but also that this immune response is associated with the induction of a protective response against *C. neoformans* infection (Upadhya et al., 2016; Upadhya et al., 2021). Therefore, we wanted to determine if vaccinating mice with KN99 cells grown in either YNB-U or YNB at pH 7 to reduce the amount of chitosan rendered the animals immune to a challenging infection with virulent KN99 cells. To vaccinate animals, we decreased the dose of HK KN99 cells grown in YNB-U to a dose equal to 10^6^ CFU because inoculation with 10^7^ CFU of HK KN99 cells grown in YNB-U is lethal (**Figure 7**). However, since KN99 cells grown in either YPD or YNB, pH 7, did not cause a major inflammatory response when administered at a dose equal to 10^7^ CFU, we chose to use this dose for vaccinating CBA/J mice. Only cells grown in YNB, pH 7, provided complete protection against a subsequent challenge infection with the virulent KN99 cells, as shown in **Figure 9A**. No protective immunity was induced when either YPD- or YNB-U-grown cells were used as a vaccine (**Figure 9A**).

Different laboratory mouse strains have been observed to elicit varying degrees of protection against *C. neoformans* infection after immunization with potential vaccine candidate strains/protein conjugates has been observed to elicit varying degrees of protection against *C. neoformans* infection in different laboratory mouse strains.. In these studies, booster vaccinations doses were found to improve protective immunity against *C. neoformans* challenge infection (Specht et al., 2017; Masso-Silva et al., 2018; Wang et al., 2019). Although the HK chitosan-defective *cda1Δcda2Δcda3Δ* mutant provided complete protection in CBA/J, 129, and A/J mouse strains, it provided only partial protection in C57BL/6 and BALB/c strains, as previously demonstrated (Upadhya et al., 2016). As a result, we wanted to see if the protective immunity induced by inoculation with HK KN99 that were previously grown on YNB, pH 7, was also effective in the C57BL/6 mouse strain. We decided to use a vaccination dose equivalent to 10^8^ CFU of KN99 cells because inoculation with 10^7^ CFU of HK KN99 cells cultured in YNB, pH 7, resulted in a subdued inflammatory response (**Figure 8**). Furthermore, rather than a single prime boost of the vaccine at –40 days relative to the challenge infection day, we gave a second group of mice a secondary booster dose of vaccination 2 weeks (–28 days) after the initial booster vaccination. These mice were challenged with 50,000 CFU of KN99 cells on day 0 and their survival was monitored. As shown in **Figure 9B**, the mice that received a single dose of vaccination had a median survival duration of 26 days, compared with 19.5 days for the unvaccinated control group (**Figure 9B**; single dose). In contrast, all mice that received a second booster dose of vaccine survived a lethal dose of KN99 challenge infection when monitored up to 60 DPI (**Figure 9B**; double dose). These findings show that growth-induced changes in the cell wall make even wild-type KN99 cells an effective potential vaccine against *C. neoformans* in a variety of laboratory mouse strains.

## Discussion

*C. neoformans* develops distinct phenotypes in a variety of media and culture conditions that are a result of the yeast cells’ external stress adaptation mechanisms. During growth, most fungal organisms are known to acidify the YNB medium. This acidification has been attributed to either sugar metabolism or the activity of the proton pump located in the membrane (Kotyk et al., 1999). Several fungal species are locked in their yeast form in an acidic medium environment, indicating that acidity has a significant impact on cell wall rearrangement (Bartnicki-Garcia and Nickerson, 1962; Odds, 1985; Szabo and Stofanikova, 2002). The acidification of the YNB medium during the growth of *C. neoformans* has been previously reported (Farkas et al., 2009). In late stationary-phase cells, growth in YNB medium has been shown to cause autolysis and significant cell wall remodeling, with secondary cell wall formation (Farkas et al., 2009). In contrast to the growth in YNB-U, when KN99 cells were grown in YPD, the pH of the medium increased to 8, three logs higher than the starting pH of 5. This could be due to the growing cells secreting a specific metabolite or molecule that affected the pH.

A direct comparison of cell wall components in *Saccharomyces cerevisiae* grown in YPD and YNB medium revealed that the amount of chitin, mannan, and β-glucan in the YNB-grown cells was significantly lower than in the YPD-grown cells (Aguilar-Uscanga and Francois, 2003). We discovered that, in *Cryptococcus*, cell growth in YNB-U greatly reduces chitosan content. We were able to produce chitosan-deficient cells simply by changing the medium composition, whereas producing chitosan-deficient mutants required the deletion of three *CDA* genes (Baker et al., 2007). Furthermore, chitosan-deficient KN99 cells produced in YNB-U medium displayed phenotypes similar to genetic mutants lacking chitosan (Baker et al., 2007). We discovered that cells grown in three different media have nearly identical amounts of chitin, as determined by the MBTH assay, indicating that the chitosan deficiency is not reflected in the amount of cellular chitin produced by the cells. One possible explanation for this is that in acidic conditions chitosan solubility increases, and *Cryptococcus* may be preventing the solubilization of chitosan by reacetylation to chitin in YNB-U-grown cells. Indeed, recombinant enzymes of several bacterial and fungal chitin deacetylases have been shown to catalyze N-acetylation of glucosamine tetramer (Hembach et al., 2017). However, caution should be exercised in considering this possibility because, in the reported study, N-acetylation of glucosamine tetramers was demonstrated at pH 7.0. Furthermore, we were unable to detect glucosamine or chitosan oligomers in the YNB-U supernatant using the MBTH assay. More sensitive and specific assays are required to confirm this observation. Despite a similar amount of chitin from a biochemical assay, the intensity of CFW staining was slightly higher in YNB-U-grown cells than in YPD-grown cells. This could be because CFW has easier access to the chitin in the cell wall of cells grown in YNB-U and/or may bind non-specifically to an unknown component in the cell wall.

One of the important findings of the present study is that chitosan biosynthesis is extremely sensitive to the composition of the growth medium. In cells grown in nutrient-limited YNB (YNB-U or YNB, pH 7) medium, the amount of chitosan in the cell wall is greatly reduced. Although CDAs deacetylate chitin to produce chitosan, we have previously shown that only the chitin synthesized by Chs3p can be deacetylated in *C. neoformans*. This is because, despite having all three *CDA* genes and chitin produced by other chitin synthases in the cell wall, the mutants *chs3Δ* and *csr2Δ* fail to produce chitosan (Baker et al., 2007). This implies that, in *C. neoformans*, only the chitin produced by Chs3p and Csr2p can be deacetylated by the CDAs. Alternatively, *C*. *neoformans* CDAs could be inactive or not expressed in the absence of either *CHS3* or *CSR2*. A variety of factors can influence the activities of either Chs3p or CDAs in cells grown in YNB-U or YNB, pH 7. As shown in **Figure 2B**, cells grown in YNB-U or YNB, pH 7, were much more sensitive to SDS than cells grown in YPD, suggesting the potential disruption of membrane homeostasis. This could have negatively influenced the enzyme activities of Chs3 or CDAs that are membrane bound. This is consistent with a previous study that showed that the membrane association of Cda2p is critical for its deacetylase activity (Gilbert et al., 2012). The inactivation of either Chs3p, Csr2p, or the CDAs in the acidic environment may be responsible for the further reduction in the amount of chitosan observed in the cells grown in YNB-U compared with those grown in YNB, pH 7. This is especially true for CDAs, which have an ideal pH range of 4.5–12 (Zhao et al., 2010).

In addition to the lack of chitosan, cells grown in YNB-U medium stain more strongly with a variety of fluorescent probes specific to cell walls. The efficient binding of WGA, Con A, and the antibody against β-1,3-glucan suggests that these molecules can easily access the binding sites in the altered cell wall of YNB-U-grown cells. This is consistent with the results of our TEM analysis of the cell wall ultrastructure. These results revealed that growth in YNB-U resulted in significant surface unmasking of cell wall components, whereas growth in YPD or YNB, pH 7, preserved PAMP masking on the yeast cell surface. Despite similar nutritional conditions, buffering the media with MOPS to a neutral pH of 7 enabled proper cell wall organization, which improved PAMP masking on yeast cell surfaces. Exposing YNB, pH 7-grown cells to acidic pH or to the supernatant of YNB-U culture medium had no effect on WGA binding. This rules out either a simple acid environment degrading the cell wall or biological activity in the YNB-U culture supernatant, such as low-pH-activated hydrolytic enzymes, causing cell wall damage. These findings imply that acidic conditions impede the proper assembly of the cell wall during yeast growth. However, once the cell wall is properly organized, as in cells grown in YNB, pH 7, they are resistant to disorganization simply by being exposed to low pH.

The increased exposure of surface-exposed PAMPs on YNB-U-grown cells resulted in their efficient recognition by the innate immune system, resulting in a major inflammatory response in the lungs. The higher exposure of WGA-specific binding sites in *C. neoformans* has been shown to activate macrophages *in vitro* and stimulate inflammatory responses *in vivo* (O’Meara et al., 2013; Esher et al., 2018). In fact, prior to infection, neutralizing the exposed chito-oligomers on the surface of yeast cells with WGA has been shown to reduce fungal virulence and dissemination to the brain (Fonseca et al., 2013). Previous studies have found that the nature of the inflammatory response produced in the lungs after infection with two separate chitosan-deficient mutants differs (Upadhya et al., 2016; Hole et al., 2020). Whereas the infection of mice with an HK *cda1Δcda2Δcda3Δ* resulted in a controlled induction of pro-inflammatory response, which was accompanied by a Th-1 type adaptive protective response, infection with an HK *chs3*Δ resulted in an uncontrolled inflammatory response, resulting in early morbidity (Upadhya et al., 2016; Hole et al., 2020). The response triggered and the phenotypes observed in our study in animals infected with YNB-U-grown cells were similar to the clinical symptoms reported during infection with the *chs3*Δ mutant. In addition to the robust release of pulmonary IL-6 and G-CSF, similar to infection with *chs3*Δ, YNB-U-grown cells also induced the expression of additional inflammatory molecules such as IL-1α, TNF-α, and the CCL class of chemokines. This suggests that, in addition to the lack of chitosan, there may be other differences in the pattern of PAMP exposure on the surface of these fungal cells, as well as differences in the physicochemical properties of various surface glycans, such as size, solubility, and tertiary structures, between *chs3*Δ and YNB-U-grown cells. Nonetheless, the robust production of IL-6 and G-CSF by YNB-U-grown cells suggests a potential involvement of neutrophils. Therefore, it will be interesting to see whether the inflammatory response triggered by YNB-U-grown cells can be prevented by the depletion of neutrophils, as seen with *chs3*Δ (Hole et al., 2020). It is interesting that, in spite of infiltration of excessive leukocytes to the lung on day 3 post infection (**Figure S3**), infection with HK KN99 cells grown in YNB, pH 7, did not cause inflammation in the lungs (**Figure 8**). The fact that these cells conferred an effective protective response suggests that vaccination with these cells did eventually induce a protective adaptive immune response. One of the most important goals of our future research would be to identify the correlates of this protective immune response.

In a murine infection model, several gene deletion mutants affecting cell wall architecture in *C. neoformans* have been shown to alter the type of host immune response and virulence (Gilbert et al., 2010; O’Meara et al., 2013; Zhai et al., 2015; Esher et al., 2018; Telzrow et al., 2022; Yang et al., 2022). This is the first report in *C. neoformans* demonstrating how the type of culture medium affects the virulence of wild-type KN99 cells. The data presented here show that minor differences in how yeast cells are grown can influence the extent of virulence and type of host immune response, particularly depending on the inoculum dose. *Cryptococcus* has been found to grow in a wide range of environments. As a result, the type of environment may potentially influence the outcome of human infection by influencing the pattern of surface-exposed PAMPs on the yeast surface. Importantly, different laboratories grow yeast cells in different methods in terms of medium type and aeration. Due to the variance in virulence described here, these differences in cell preparation protocols may influence the results of *in vitro* and *in vivo* host–pathogen interaction studies.

Although inoculation with 10^6^ CFU of YNB-U grown HK KN99 did not kill mice, it did not induce protective immunity (**Figure 9A**). Protective immunity was not induced with YPD-grown HK cells either. Chitosan levels in YNB, pH 7-grown cells were found to be half of those found in YPD-grown cells. It has previously been demonstrated in a variety of inbred mouse strains that, when used as a vaccine, a HK mutant of *C. neoformans* lacking chitosan in the cell wall, induced protective immunity (Upadhya et al., 2016). We do not yet understand the nature of these protective mechanisms or how, if at all, chitosan levels in the cell wall contribute to protection. Various mutants of *C. neoformans* have been shown to induce protective immunity when used as a vaccine (Wormley et al., 2007; Rella et al., 2015; Zhai et al., 2015; Masso-Silva et al., 2018). Many of the mutant strains have gene mutations that affect the cell wall or cell membrane integrity, with the exception of the H99γ strain, which was designed to generate murine interferon-γ. Similarly, when introduced into animals or presented to immune cells *in vitro*, different mutants of *C. neoformans* that regulate cell wall biogenesis have been shown to cause abnormal host inflammatory responses (O’Meara et al., 2013; Esher et al., 2018). Our findings show that protective immunity can be induced with YNB, pH 7-grown HK cells even in the absence of a significant inflammatory response in the lung during vaccination. Our future goal is to determine which component of *C. neoformans* is solely responsible for inducing protective immunity without causing unwanted host damage in order to develop safe and effective vaccines against this fungal infection. This study’s findings offer a novel tool for distinguishing between fungus-specific factors that elicit a harmful host immune response and those that are only minimally required for inducing protective immunity.

## Materials and methods

### Fungal strains and media

*C. neoformans* KN99-α was used for all the experiments. Strains were grown in YPD broth (1% yeast extract, 2% Bacto peptone, and 2% dextrose) or on YNB-U (0.67% yeast nitrogen base without amino acids [Difco catalog # 291940] containing 2% dextrose) or YNB, pH 7 (YNB medium adjusted to a pH of 7 with 50 mM MOPS [3-morpholinopropane-1-sulfonic acid]). In all the experiments, yeasts were grown in a 250-mL polycarbonate Erlenmeyer flask (Corning, NY, USA) containing 50 mL of the respective medium at 30°C in a shaking incubator at 300 rpm for 48 h.

### Measurement of cell wall chitosan with 3-methyl-2-benzothiazolinone hydrazone

Cell wall chitosan was measured as described previously (Upadhya et al., 2017; Upadhya et al., 2018). In brief, 10 mL of cell culture, grown as above in the respective medium for 48 h, was harvested by centrifugation in pre-weighed 15-mL Falcon tubes. The cell pellet was washed once with PBS and lyophilized. Lyophilized cells were weighed, and the dry weight of the cell pellet was recorded. A lyophilized cell pellet was resuspended in 6 mL of PBS, and the cell pellet was vortexed to generate a uniform cell suspension devoid of cell clumps. KOH was then added to a final concentration of 6% (w/v), and the suspension was incubated at 80°C for 1 h with intermittent vertexing. After 1 h, cell pellet was collected by centrifugation (3,500 × g) and washed three times with 10 mL of PBS every time. Each time, the cell pellet was vortexed s to get rid of any cell clumps. After the final wash, the cell pellet was suspended in PBS at 10–20 mg dry weight/mL. This cell suspension was then sonicated using a probe sonicator (a Thermo Fisher Sonic Dismembrator, model 300, attached to a GraLab model 451 high-accuracy digital electronic timer) for five cycles of 60 s. A 0.1-mL aliquot of the alkali-treated material was used for the chitosan measurement. Frist 100 µL of 2 M HCl was added to acidify the sample. Then 400 µL of sodium nitrite was added and vortexed and the reaction was incubated at 25°C for 15 min. Next, 200 µL of 12.5% ammonium sulfamate was added dropwise to neutralize the unreacted sodium nitrite. Care should be taken to prevent sample loss due to effervescence. After 5 min incubation at 25°C, 200 µL of freshly prepared MBTH solution (0.25% w/v) was added and the sample was incubated at 37°C for 30 min. At the end of the incubation, 200 µL of 0.5% ferric chloride was added and the sample was incubated further at 37°C for 15 min after mixing. At the end of the incubation, samples were centrifuged at 12,000 × g for 10 min. An aliquot of 200 µL of the supernatant was used for measuring the absorbance at 650 nm. d-glucosamine was used as the standard in the concentration range of 10–100 nmol. The amount of chitosan was expressed as nmol of glucosamine per milligram dry weight of the cellular material.

### Quantification of cell wall-specific molecules after staining with various cell wall-specific fluorescent stains/molecules by fluorescence-activated cell sorting and fluorescence imaging

Cells were grown in the respective medium as described above and were collected by centrifugation at 3,500 × g for 8 min at 4°C. For staining with Calcofluor White (CFW), Alexa Fluor 488-conjugated wheat germ agglutinin (WGA; Invitrogen™ W11261), concanavalin A fluorescein isothiocyanate (FITC)-conjugated Con A (Sigma, catalog no. C7642), β-(1,3)-glucan antibody 2G8 (ab233743; Abcam, USA), and Alexa 555-conjugated mouse secondary antibody (A32727; Invitrogen) were used. Cells were fixed with 4% paraformaldehyde for 30 min on ice. Fixed cells were washed twice with PBS and blocked with 2% bovine serum albumin (BSA; Sigma A7906) at 30°C for 30 min in a nutating mixer. CFW, WGA, and ConA were used at 5 µg/mL, 100 µg/mL, and 50 µg/mL respectively. Cells were incubated with the stains on ice for 30 min in the dark. Stained cells were washed with PBS three times and spotted on the slides. Slides were imaged using an Olympus microscope using a 63× objective. For microscopy, WGA was detected using the Green Fluorescent Protein (GFP) filter settings, and CFW was detected using the 496-diamidino-2-phenylindole (DAPI) settings in a Zeiss Axio Imager M2 equipped with a Hamamatsu Flash4.0 Complementary Metal-Oxide-Semiconductor (CMOS) camera. Flow cytometry data were acquired using a BD LSRFortessa cell analyzer. The WGA signal was detected using a 488 nm laser, and the CFW signal was detected using a 405 nm laser. Flow cytometry data were analyzed with FlowJo version 10.0 software. Representative histogram data are shown in **Figures 3E–H**.

### Cell wall and temperature sensitivity growth assays

Cultures grown for 48 h in the indicated media were diluted to OD_650_ = 1.0, and 5-μL portions of the 10-fold serially diluted cell suspensions were spotted on YPD plates containing appropriate stressors and incubated for 3 days at 30°C. For cell wall stress, YPD agar plates were supplemented with either SD (0.015%) or CFW (Fluorescent Brightener 28, F-3543; Sigma, USA; 1.5 mg/mL). To test for temperature sensitivity, YPD plates were incubated at 25°C and 39°C for 3 days.

### Capsule analysis

Yeast cells were initially grown in the respective medium for 48 h as described above. Cells were collected and washed twice with sterile PBS (pH 7.4). Cell pellets were resuspended in 50 µL of India ink (American Master Tech Scientific, ref. no. STIIN25). Ten microliters of the cell suspension was used for imaging.

### Analysis of melanin production

KN99 cells grown in the respective medium for 48 h were collected, washed with PBS, and resuspended at 5 × 10^7^ cells/mL in 2 mL of glucose-free asparagine medium (1 g/L l-asparagine, 0.5 g/L MgSO_4_·7H_2_O, 3 g/L KH_2_PO_4_, and 1 mg/L l-thiamine) plus 1 mM L-3,4-dihydroxyphenylalanine (l-DOPA). Cells were shaken at 30°C for 24 h in a Falcon tube. At the end of incubation, cells were collected by centrifugation, and the tube with the pellet and the supernatant was photographed.

### Transmission electron microscopy

For ultrastructural analyses, cells were fixed in 2% paraformaldehyde/2.5% glutaraldehyde (Polysciences, Inc., Warrington, PA, USA) in 100 mM sodium cacodylate buffer, pH 7.2, for 2 h at room temperature and then overnight at 4°C. Samples were washed in sodium cacodylate buffer and post-fixed in 1% osmium tetroxide (Polysciences, Inc.) for 1 h at room temperature. Samples were then rinsed extensively in dH_2_O prior to *en bloc* staining with 1% aqueous uranyl acetate (Ted Pella, Inc., Redding, CA, USA) for 1 h. Following several rinses in dH_2_O, samples were dehydrated in a graded series of ethanol and embedded in Eponate 12 resin (Ted Pella, Inc.). Sections of 95 nm were cut with a Leica Ultracut UC7 ultramicrotome (Leica Microsystems, Inc., Bannockburn, IL, USA), stained with uranyl acetate and lead citrate, and viewed on a JEOL 1200 EX transmission electron microscope (JEOL USA, Inc., Peabody, MA, USA) equipped with an AMT 8-megapixel digital camera and AMT Image Capture Engine V602 software (Advanced Microscopy Techniques, Woburn, MA, USA).

### Animal infection and fungal burden assays

Pulmonary infections and virulence assays were performed as described previously (Missall et al., 2004b). *C. neoformans* KN99 was grown in 50 mL of the indicated medium in a 250-mL flask shaken at 300 rpm for 48 h. Cells were collected by centrifugation at 3,000 × g for 10 min. The cell pellet was washed twice with endotoxin-free PBS and adjusted to 1 × 10^6^cells/mL in PBS. For intranasal inoculations, female CBA/J mice, approximately 5–6 weeks old (Jackson Laboratories, Farmington, CT, USA), were first anesthetized with an intraperitoneal injection (200 μL) of a ketamine (8 mg/mL)/Dexdomitor (Zoetis, Parsipanny, NJ, USA) (0.05 mg/mL) mixture, then weighed and intranasally infected with 50 μL of the cell suspension. After 10 min they were injected intraperitoneally (200 μL; 0.25 mg/mL) with the reversal agent atipamezole (Antisedan). The viability of the inoculated KN99 cells was confirmed after plating the serially diluted inoculum onto YPD plates and incubating them at 30°C for 72 h to enumerate CFU. Mice were monitored daily, and those showing any signs of morbidity (weight below 80% of pre-inoculation weight, extension of the cerebral portion of the cranium) were sacrificed by CO_2_ asphyxiation and cervical spine dislocation. All animal protocols were reviewed and approved by the Washington University School of Medicine Animal Care and Use Committee (IACUC protocol # 20–0474) and Duke University Institutional Animal Care and Use Committee (protocol # A113-22-06). Animal survival differences were determined by Kaplan–Meier analysis. The fungal burden was assessed by excising organs, homogenizing them in PBS, and plating the diluted homogenate on YPD agar for enumerating CFUs. Organ burden was analyzed by the Kruskal–Wallis test with Dunnett’s multiple comparison *post hoc* test for each day post infection.

### Lung histopathology studies

Mice were euthanized on day 3 post infection. The lungs were initially perfused intracardially with PBS. PBS-perfused lungs were then inflated with 10% neutral-buffered formalin, and the trachea was tied off with a ligature. The inflated lung was removed *en bloc* with the heart and stored in 10% neutral-buffered formalin solution for 48 h. After 48 h, the heart tissue was separated and the whole lung was placed in a cassette and submitted to HistoWiz, Inc. (Brooklyn, NY, USA), for histology by following recommended procedures. Samples were processed, embedded in paraffin, cut into 4-µm sections, and stained with hematoxylin and eosin (H&E). After staining, sections were dehydrated and film coverslipped using a Tissue Tek-Prisma and Coverslipper (Sakura USA, Torrance, CA, USA). Whole-slide scanning (40×) was performed on an Aperio AT2 instrument (Leica Biosystems, Wetzlar, Germany).

### Pulmonary cytokine measurement

The Bio-Plex protein array system (Bio-Rad Laboratories, Hercules, CA, USA) was used to look at the levels of cytokines in lung tissue. Briefly, lung tissue was excised at the indicated number of days post infection, and homogenized in 2 mL of ice-cold PBS containing 1× Pierce protease inhibitor cocktail (Thermo Fisher Scientific, Rockford, IL, USA). After homogenization of the lung tissue, Triton X-100 was added to a final concentration of 0.05%, and the samples were clarified by centrifugation and stored at –80°C. The supernatant fractions from the pulmonary homogenates were then assayed using the Bio-Plex Pro Mouse Cytokine 23-Plex (Bio-Rad Laboratories) by following the protocol supplied with the kit. In each well, 50 μL of lung homogenate (1:1 diluted) was analyzed.

## Data availability statement

The original contributions presented in the study are included in the article/**Supplementary Material**. Further inquiries can be directed to the corresponding authors.

## Ethics statement

The animal study was reviewed and approved by the Washington University Institutional Animal Care and Use committee (protocol # 20-0474) and the Duke University Institutional Animal Care and Use Committee (protocol # A113-22-06).

## Author contributions

RU: conceptualization, methodology, investigation, validation, formal analysis, visualization and writing original draft, review, and editing. WL: conceptualization, methodology, investigation, validation, formal analysis, writing review, and editing. CH: conceptualization, methodology, investigation, validation, formal analysis, writing review, and editing. JV: conceptualization, methodology, investigation, validation, formal analysis, writing review, and editing. JL: conceptualization, methodology, formal analysis, visualization, writing review, editing, funding acquisition, and project administration. All authors contributed to the article and approved the submitted version.
