# Supplementary material for: Cell wall composition in Cryptococcus neoformans is media dependent and alters host response, inducing protective immunity
Source: Front Fungal Biol. 2023 May 12;4:1183291. doi: 10.3389/ffunb.2023.1183291 (PMC10399910; doi:10.3389/ffunb.2023.1183291)
Supplement: Supplementary file 1 [file Presentation_1.pptx]

## Slide 1
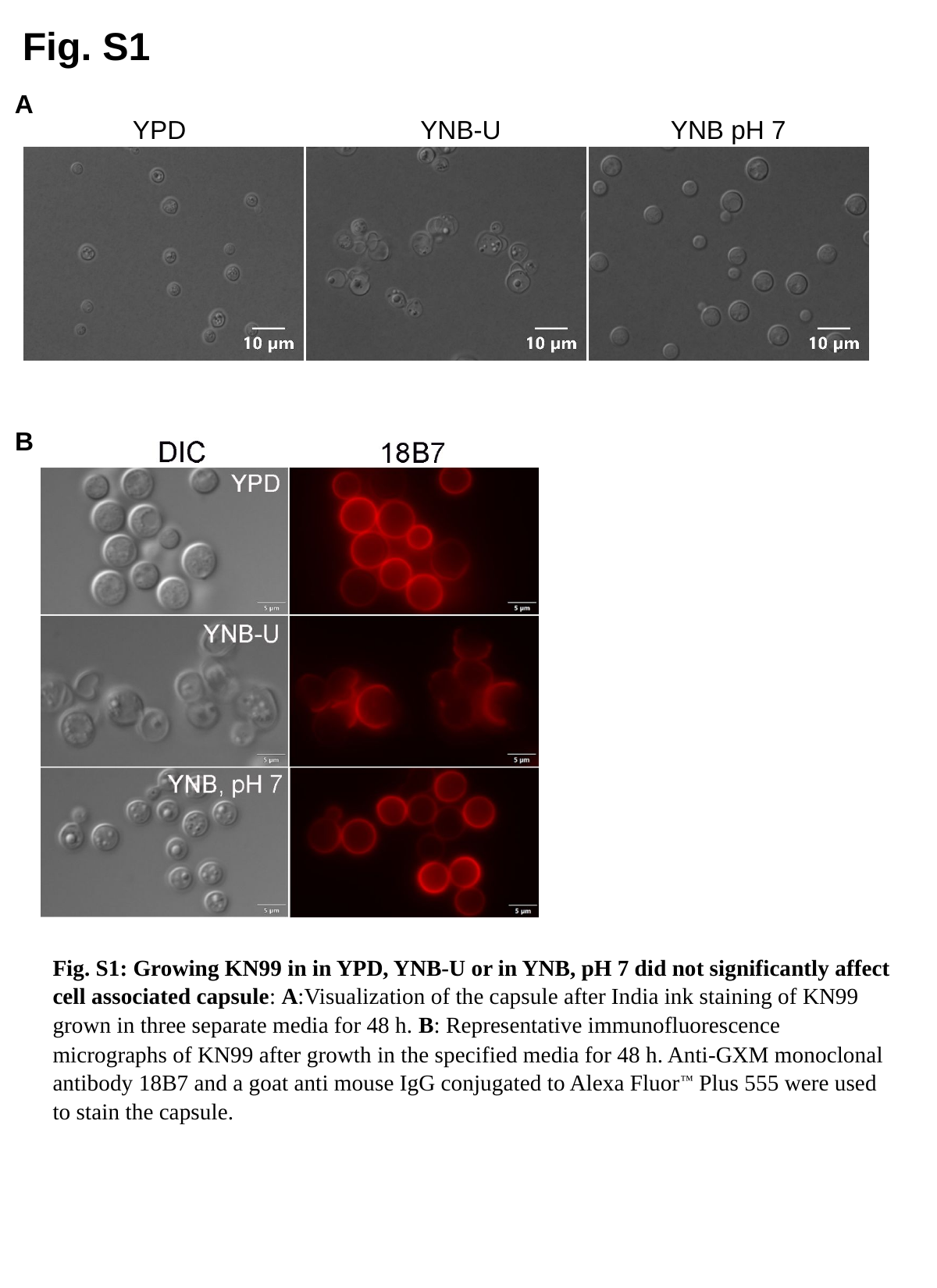

Fig. S1
A
YPD
YNB-U
YNB pH 7
B
Fig. S1: Growing KN99 in in YPD, YNB-U or in YNB, pH 7 did not significantly affect cell associated capsule: A:Visualization of the capsule after India ink staining of KN99 grown in three separate media for 48 h. B: Representative immunofluorescence micrographs of KN99 after growth in the specified media for 48 h. Anti-GXM monoclonal antibody 18B7 and a goat anti mouse IgG conjugated to Alexa Fluor™ Plus 555 were used to stain the capsule.

## Slide 2
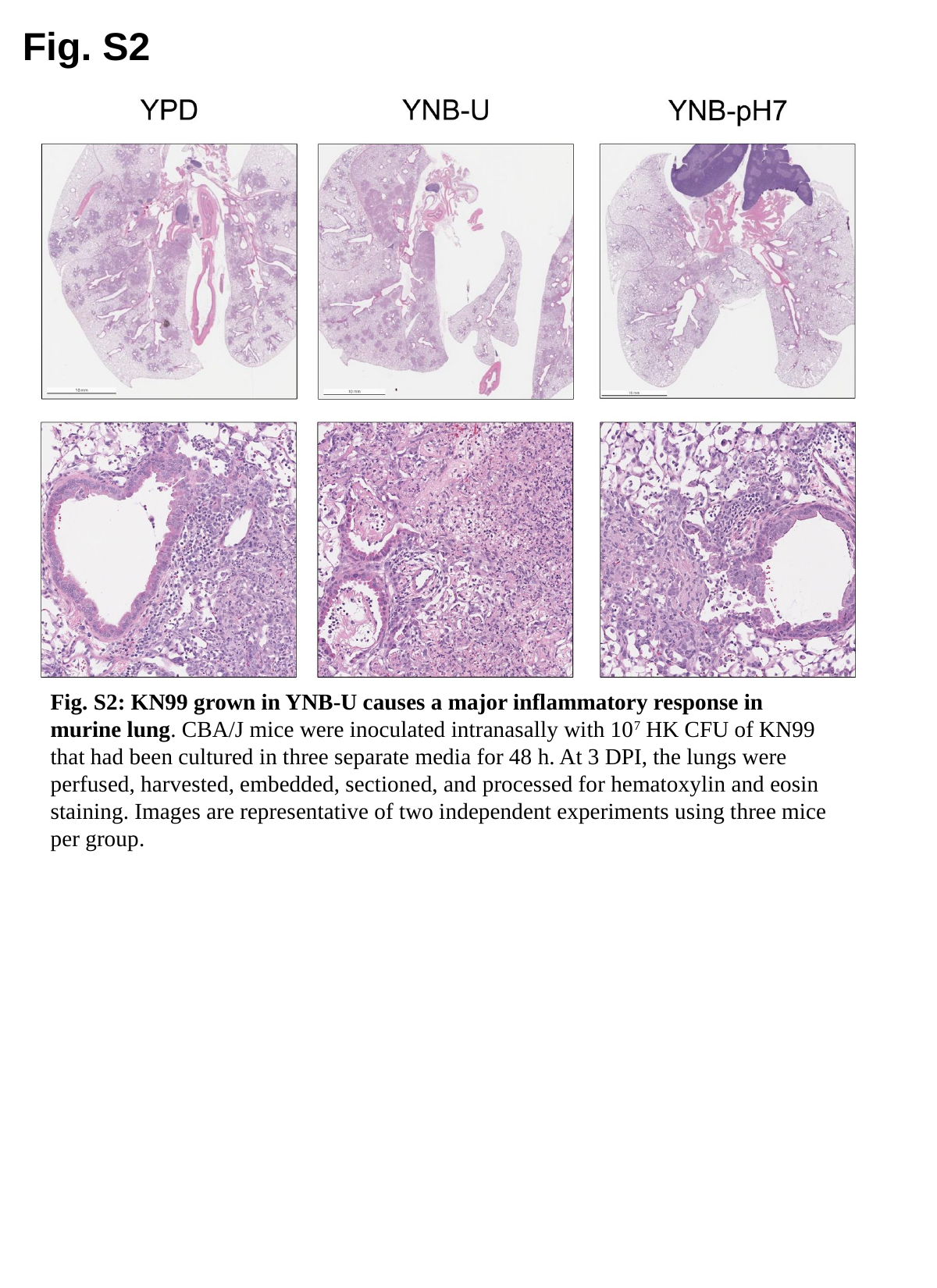

Fig. S2
Fig. S2: KN99 grown in YNB-U causes a major inflammatory response in murine lung. CBA/J mice were inoculated intranasally with 107 HK CFU of KN99 that had been cultured in three separate media for 48 h. At 3 DPI, the lungs were perfused, harvested, embedded, sectioned, and processed for hematoxylin and eosin staining. Images are representative of two independent experiments using three mice per group.

## Slide 3
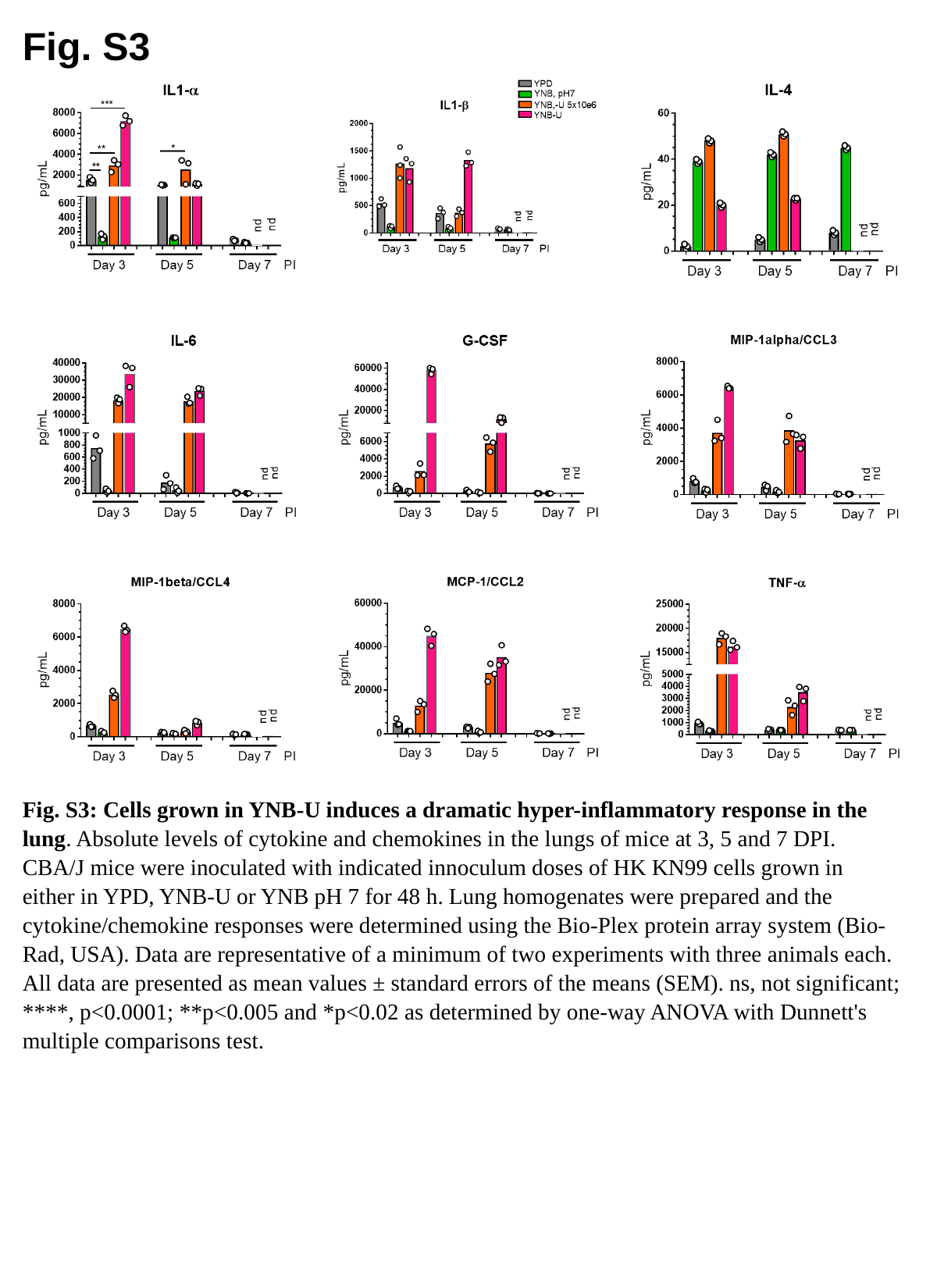

Fig. S3
Fig. S3: Cells grown in YNB-U induces a dramatic hyper-inflammatory response in the lung. Absolute levels of cytokine and chemokines in the lungs of mice at 3, 5 and 7 DPI. CBA/J mice were inoculated with indicated innoculum doses of HK KN99 cells grown in either in YPD, YNB-U or YNB pH 7 for 48 h. Lung homogenates were prepared and the cytokine/chemokine responses were determined using the Bio-Plex protein array system (Bio-Rad, USA). Data are representative of a minimum of two experiments with three animals each. All data are presented as mean values ± standard errors of the means (SEM). ns, not significant; ****, p<0.0001; **p<0.005 and *p<0.02 as determined by one-way ANOVA with Dunnett's multiple comparisons test.
